# Limited freshwater cap in the Eocene Arctic Ocean

**DOI:** 10.1038/s41598-019-40591-w

**Published:** 2019-03-12

**Authors:** Lisa A. Neville, Stephen E. Grasby, David H. McNeil

**Affiliations:** 1 Geological Survey of Canada, Natural Resources Canada, Calgary, Alberta T2L 2A7 Canada; 2 AGAT Laboratories, Calgary, Alberta T2E 7J2 Canada

## Abstract

Remains of the freshwater fern *Azolla*, found in Eocene (~50 Ma ago) sediments in the modern central Arctic Ocean, have been used to suggest that seasonal freshwater caps covered the entire Arctic Ocean during that time, with significant impact on global ocean circulation and climate. However, these records are located on the Lomonosov Ridge, which during the Eocene was a continental fragment barely rifted from Eurasia, separating the smaller Eurasian Basin from the much larger Amerasian Basin to the west. As such, the Lomonosov Ridge does not necessarily record environmental conditions of the broader Arctic Ocean. We tested the hypothesis of freshwater caps by examining sediment records from the western Amerasian Basin. Here we show that in the larger Amerasian Basin the *Azolla* event is associated with marine microfauna along with allochthonous (terrestrially sourced) organic matter. We propose that *Azolla* events are related to an increased hydrologic cycle washing terrestrially sourced *Azolla*, and other organics, into the Arctic Ocean. If freshwater caps did occur, then they were at best restricted to the small Eurasian Basin and would have had a limited impact on Eocene global climate, contrary to current models.

## Introduction

**Figure 1:**
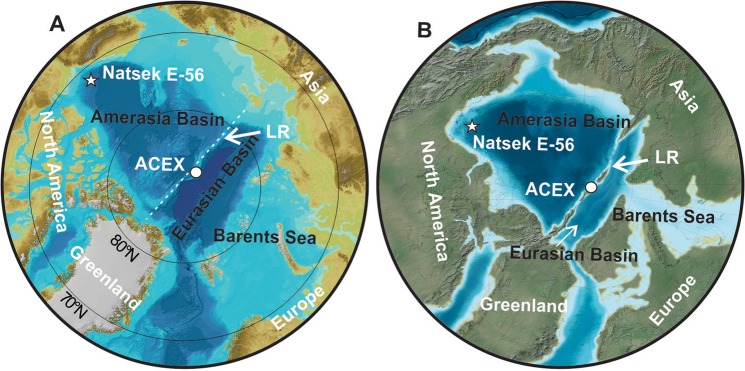
Images showing Modern and Eocene Arctic Ocean. (**A**) Modern bathymetric chart for the Arctic Ocean^37^. (**B**) Eocene palaeoreconstruction^1,2^. White star marks location of Natsek E-56 well, LR = Lomonosov Ridge, white dot marks the ACEX drilling location.

The late Paleocene and early Eocene (47.8 to 59.2 Ma time period) provides an analogue to understand impacts of modern climate warming, particularly at high latitudes. During this time the Arctic experienced extremely warm conditions, including rainforests inhabited by alligators and giant tortoises^1,2^ with mean annual air temperatures reaching ~15 °C^3–5^. However, the palaeoenvironment of the Eocene Arctic Ocean has been largely inferred from only one locality, the ACEX cores from the Lomonosov Ridge^6–8^. At this location the ‘*Azolla* event’ was defined by thousands of *Azolla* containing laminae observed in mid Eocene sediments over ~800 kyr^6^. The large numbers of the free-floating freshwater *Azolla* fern found at the ridge are suggested to be a product of fern growth on surface freshwater layers, with floating mats covering the entire Arctic Ocean^9^. The co-occurrence of marine dinocysts, diatoms, silicoflagellates and ebridians was explained by Arctic Ocean surface waters being only seasonally fresh^8^. These Eocene sediments of the Lomonosov Ridge are purported to reflect mid-Artic Ocean conditions. This, however, is not consistent with plate tectonic models. The Lomonosov Ridge from which the ACEX cores were collected is a seafloor feature more than 1500 km long and 30–80 km wide, rising 3 km above the abyssal plains^10^. The ridge rifted from Eurasia ~55 Ma, opening the Eurasia Basin^11,12^. Rifting formed a shallow sill^8,10^ separating the older and much larger Amerasian Basin from the younger, smaller and shallower Eurasian basin^12^ (Fig. 1). Micropaleontological data indicate that marine sediments on the Lomonosov Ridge had an epipelagic depth (0–200 m) throughout the Paleogene^13^. This is thought to relate to heat flow from the Nansen-Gakkel seafloor spreading centre, and voluminous magmatism^14^, as well as far-field in-plane stress that kept the Lomonosov Ridge shallow, if not elevated above sea level in places, until the Miocene^1,15,16^. An exposed ridge has even been suggested to have acted as a temporary land bridge during the Eocene that stimulated the movement of plants and animals^1^. Thus it remains unclear if the marine sediments from the ACEX core of the Lomonosov Ridge truly represent open Arctic Ocean conditions, or did the ridge itself act as a sill that formed a restricted Eurasian sub-basin. To test the palaeoceanographic model of the entire Arctic Ocean containing fresh surface waters, with extensive floating *Azolla* mats, we examined coeval records from the dominant Amerasian Basin, from a well drilled in the Beaufort Mackenzie Basin (BMB) – western Artic.

## Results

### *Azolla* event in the western Arctic

**Figure 2:**
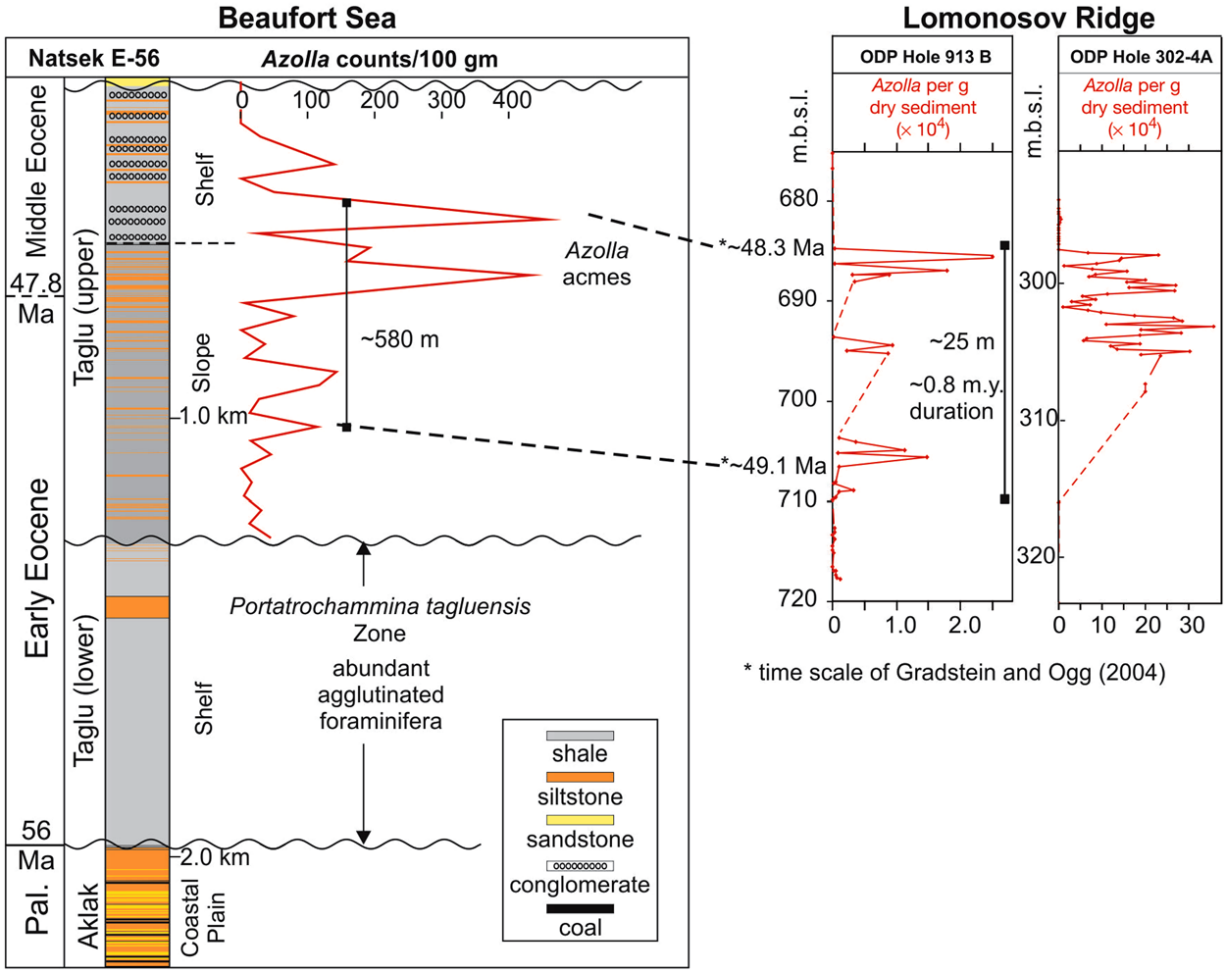
Natsek E-56 stratigraphy and lithology with corresponding ages, and micropaleontological characteristics.

**Figure 3:**
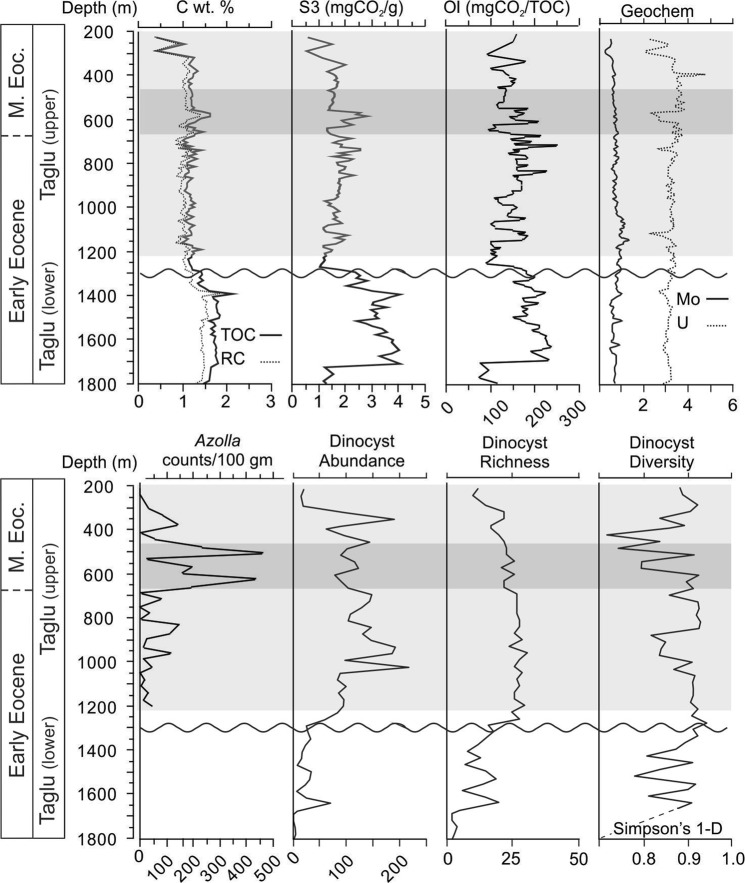
Micropalaeontological and rock-eval data for the Natsek E-56 well. TOC = total organic carbon, RC = residual carbon, OI = oxygen index. Depth represents true vertical depth. (kelly bushing, true vertical depth). Horizontal grey box represents the *Azolla* acme (counts tabulated from megaspores and massulae).

The late Cretaceous–Cenozoic BMB contains 14 km of sediment infill in a rapidly subsiding deltaic and marine environment. We examined the sediment record from a well named ‘Dome Pacific *et al*. PEX Natsek E-56′ (herein Natsek E-56) (Fig. 1) located in the southwestern part of the BMB (69°45′21.46″N; 139°44′3458″W, Northwest Territories, Canada). Natsek E-56 preserves the early to mid Eocene Taglu Sequence (including the *Azolla* event; Fig. 2)^16,17^ that consists of weakly consolidated silty to pebbly mudstone. The well represents an expanded section characterized by rapid rates of sedimentation that is at least 9 times greater than the Lomonosov Ridge, with 2 km of deposition during the Eocene^17^ (Fig. 2). The depositional environment changed significantly across an intra-Taglu unconformity, from outer shelf (mesohaline marine micropalaeontological character) to slope/shelf (with marine dinoflagellate cysts and *Azolla* present)^18,19^ (Table 1, Figs 2 and 3).

**Table 1:** Summary results for Natsek E-56 of Rock-Eval analyses (pyrolyzed carbon (PC); residual carbon (RC); total organic carbon (TOC); hydrogen index (HI); oxygen index (OI); mineral carbon (MinC)) and carbon isotope organic carbon (δ^13^C_org_).

Sequence		Depth m	S2 mgHC/g	S3 mgCO_2_/g	Tmax °C	PC wt.%	RC wt.%	TOCwt.%	HI mgHC/TOC	OI mgCO_2_/TOC	MinC wt.%	δ^13^C –_org_ ‰
Taglu	**Entire Taglu**	**1950-229**	**0.80**	**2.19**	**417.47**	**0.18**	**1.15**	**1.33**	**57.03**	**161.03**	**0.34**	**−28.61**
	*Azolla* event – mid Taglu	1226-229	0.57	1.72	411.67	0.15	1.02	1.17	47.92	148.32	0.34	−28.55
	Lower Taglu	1226–1950	1.22	3.05	428.17	0.23	1.39	1.62	73.85	184.50	0.35	−28.71
Aklak	**Entire Aklak**	**1950–2226**	**10.00**	**5.49**	**428.70**	**1.12**	**7.02**	**8.14**	**108.15**	**93.93**	**0.82**	**−27.12**

The results are further subdivided at the intra-Taglu unconformity separating the lower from mid Taglu Sequence. The average of the results for the entire sequence are in bold. The full Rock-Eval data set for Natsek E-56 are reported elsewhere^32^. HI = Hydrogen index (S2 × 100/TOC), OI = Oxygen index (S3 × 100/TOC).

The lower Taglu Sequence contains a well-developed circum-Arctic early Eocene benthic agglutinated foraminiferal assemblage assigned to the *Portatrochammina tagluensis* Zone (Fig. 2). Foraminifera disappear entirely above the unconformity in the mid Taglu Sequence, replaced by a diverse assemblage of pollen, remains of the freshwater fern *Azolla* (preserved as megaspores and massulae (Fig. S1)), as well as dinocysts and other microfossils. The *Azolla* occurrences consist of two major peaks, one in the upper slope sediments and the other in the outer shelf (Fig. 2). The *Azolla* acmes in Natsek E-56 are coeval with the *Azolla* event on the Lomonosov Ridge in the earliest mid Eocene (onset 50 Ma)^6^, with similar peaks displayed between both localities. However, our measured average abundance of *Azolla* plant parts (85 remains per 100 g of dry sediment; maximum 460 remains/100 g of dry sediment at 521 m) (Fig. 3) is significantly lower than the 5–30 × 10^6^ remains per 100 g of dry sediment observed in ACEX^6^. Our Rockeval data also show that the *Azolla* event laminae in Natsek E-56 have low organic content (Total Organic Carbon (TOC) = 1.17 wt.%). Rockeval parameters that indicate organic matter type (i.e. marine algae versus terrestrial organics) show high RC, S3, and OI indices (RC = 1.02 wt.%, S3 = 1.72 mgCO_2_/g, OI = 148.32 mgCO_2_/TOC) (Fig. 3, Table 1). These results are characteristic of lignin and cellulose plants, indicating that the organic matter that occurs in association with the *Azolla* abundance peaks is composed primarily of terrestrial organics, including plants and woody material.

Redox sensitive elements Mo and U have concentrations consistent with post-Archean average shale (PAAS)^20^, throughout the studied interval of Natsek E-56. These redox sensitive elements are normally enriched in anoxic sediments^21^, thus the average concentrations along with low TOC values is inconsistent with a strongly stratified anoxic environment of the ACEX core. However, the loss of benthic species does indicate low oxygen conditions, suggesting an overall disoxic rather than fully anoxic basin.

### Co-occurring *Azolla* and marine microfauna

We observed a rich assemblage of dinocysts above the mid-Taglu unconformity (1250 m) that indicate *Azolla*-containing laminae were deposited under slightly less than normal marine shelf conditions (Figs 3, S2, Table S1). Dinocysts included the prevalence of *Phthanoperidinium, Cordosphaeridium, Hystrichosphaeridium, Glaphyrocysta*, and wetzelielloids, along with the sparsity of offshore genera such as *Operculodinium* and *Nematosphaeropsis*, suggesting an inner to outer neritic (shallow marine environment) character^22^. Similar marine dinocyst assemblages were also observed with the *Azolla* event in the Labrador-Baffin Seaway^23^. At the Lomonosov Ridge, however, only *Phthanoperidinium* and *Senegalinium*, low-salinity tolerant genera, were associated with *Azolla* blooms^22^. In our samples, the co-occurrence of *Azolla*, which cannot tolerate salinities higher than 1–1.6‰, and marine dinocysts in Natsek E-56 is consistent with ACEX core observations of a mixed fresh/saline water fauna. However, organic matter that co-occurs with both marine fauna and *Azolla* in Natsek E-56 has an allochthonous origin. We argue then that this is more consistent with terrestrial material (organic matter and *Azolla*) being washed into a marine environment, rather than the model of *Azolla* blooming *in situ* in seasonal freshwater caps^7^. This simpler interpretation is consistent with the lack of evidence for a strongly stratified anoxic water body and alleviates the physical and biological constraints of forming and maintaining seasonal freshwater caps with floating *Azolla* mats on the Arctic Ocean surface^6^ as discussed below.

### Constraints on forming a freshwater lens and floating mats

Modern *Azolla* ferns only float freely on the surface of quiescent freshwater, such as ponds and canals in tropical, subtropical, and warm temperate regions^6,24^. They are not found on larger water bodies exposed to significant wave action that breaks up mats; it would thus be particularly challenging for a floating fern to cover the ~11.4 × 10^6^ km^2^ Arctic Ocean^25^. Given the size and annual modern freshwater flux (~8500 km^3^)^26^ the Arctic Ocean could only develop a seasonal freshwater cap of ~0.75 m. Even if increased Eocene cyclone activity^27^ led to river flow that was a maximum of twice as high as modern (based on estimates of doubling rainfall^28^, but not accounting for increased evapotranspiration) the annual freshwater cap would not exceed 1.5 m, well within the fair-weather mixing zone and storm wave base down to 5 m^29^. The same increased frequency and intensity of cyclones in the Eocene Arctic and northward shift in storm tracks would have further exacerbated mixing^26,30^, making formation and maintenance of a freshwater cap in the central Arctic Ocean highly improbable. After annual die-off, due to lack of winter sunlight, Arctic Ocean mats would require entirely vegetative reproduction in tolerable bottom water salinities not found in marine environments. Thus, even with a freshwater cap, *Azolla* would not have germinated in the Arctic Ocean.

## Discussion

We do not attempt here to re-interpret ACEX data, and allow that the interpretation of a seasonal freshwater lens from that location may still be valid. However, based on the differences between the BMB and Lomonosov Ridge records, it is possible that a subaerial ridge, or submerged sill, separated the Arctic Ocean into two distinctive bodies of water creating differing depositional environments. The high *Azolla* accumulation observed in the ACEX core could at best be a localized phenomenon restricted to the newly formed Eurasia Basin, that did not represent overall dominant Arctic Ocean conditions. The presence of *Azolla* along with marine microfauna and terrestrial organic matter in the BMB more likely reflects the change to warmer Eocene temperatures and an increased hydrological system leading to enhanced mass transport of terrestrial material, including freshwater fern debris, from freshwater bodies surrounding the Amerasian Basin. If correct, our alternative interpretation has significant implications for models of the transition from a global greenhouse climate towards the modern icehouse being triggered by Arctic Ocean wide growth of *Azolla*^9,31^. Factoring the size of the basin, it was suggested that atmospheric carbon dioxide as high as 2500 to 3500 ppm was reduced by half after the *Azolla* Event^30^, and that growth of *Azolla* contributed to at least 40% of carbon drawdown via net carbon fixation and subsequent sequestration^29^. Our results suggest *Azolla* growth was significantly more limited than these models and do not support such levels of CO_2_ drawdown. Other mechanisms should be explored to explain amelioration of Eocene hothouse conditions.

## Methods

Cuttings and duplicate samples (n = 195 and n = 5, respectively) were collected from the Natsek E-56 well between 229–2226 m and prepared for analysis at the Geological Survey of Canada (Calgary). The handpicked samples were washed lightly with tap water to remove drilling mud then oven dried at ~35 °C for ~24 hours before being powdered. Aliquots of the powdered samples were subjected to the below analysis. Additional details are published elsewhere^32^.

Rock-Eval 6 pyrolysis was conducted at the Geological Survey of Canada (Calgary) on a Rock-Eval 6 Turbo device following the Basic Method^33,34^. Total organic carbon (TOC) content and Rock-Eval parameters were determined on bulk ground samples to provide information on organic carbon source, hydrocarbon source-rock potential, presence of hydrocarbons, and thermal maturity. Detailed data are presented elsewhere^32^.

Samples were prepared, processed and analyzed for micropalaeontological content at the Geological Survey of Canada (Calgary), following the standard methods for breaking down samples^35^. The processed residues were picked for all microfossils and the microslides are stored in the collections of the Geological Survey of Canada, Calgary (GSCC). Foraminifera were identified using standard taxonomy^36^. Palynological data was obtained from Dolby (2011)^22^ and interpreted by Dr. R.A. Fensome (Geological Survey of Canada – Atlantic).

## Supplementary information

Supplementary Information

## Data Availability

Data used in this study are available for download in Geological Survey of Canada. Open File Report 7949 as well as published data in Dolby (2011)^22^.
