# Supplementary material for: Limited freshwater cap in the Eocene Arctic Ocean
Source: Sci Rep. 2019 Mar 12;9:4226. doi: 10.1038/s41598-019-40591-w (PMC6414537; doi:10.1038/s41598-019-40591-w)
Supplement: Supplementary file 1 — Supplementary Information [file 41598_2019_40591_MOESM1_ESM.docx]

**Limited freshwater cap in the Eocene Arctic Ocean**

Lisa A. Neville, Stephen E. Grasby, David H. McNeil

**Supplementary Information**

**Fig. S1.** Age model, stratigraphy, unconformities, and microfossils for Natsek E-56. The geological time scale used is based on Gradstein and Ogg (2012).


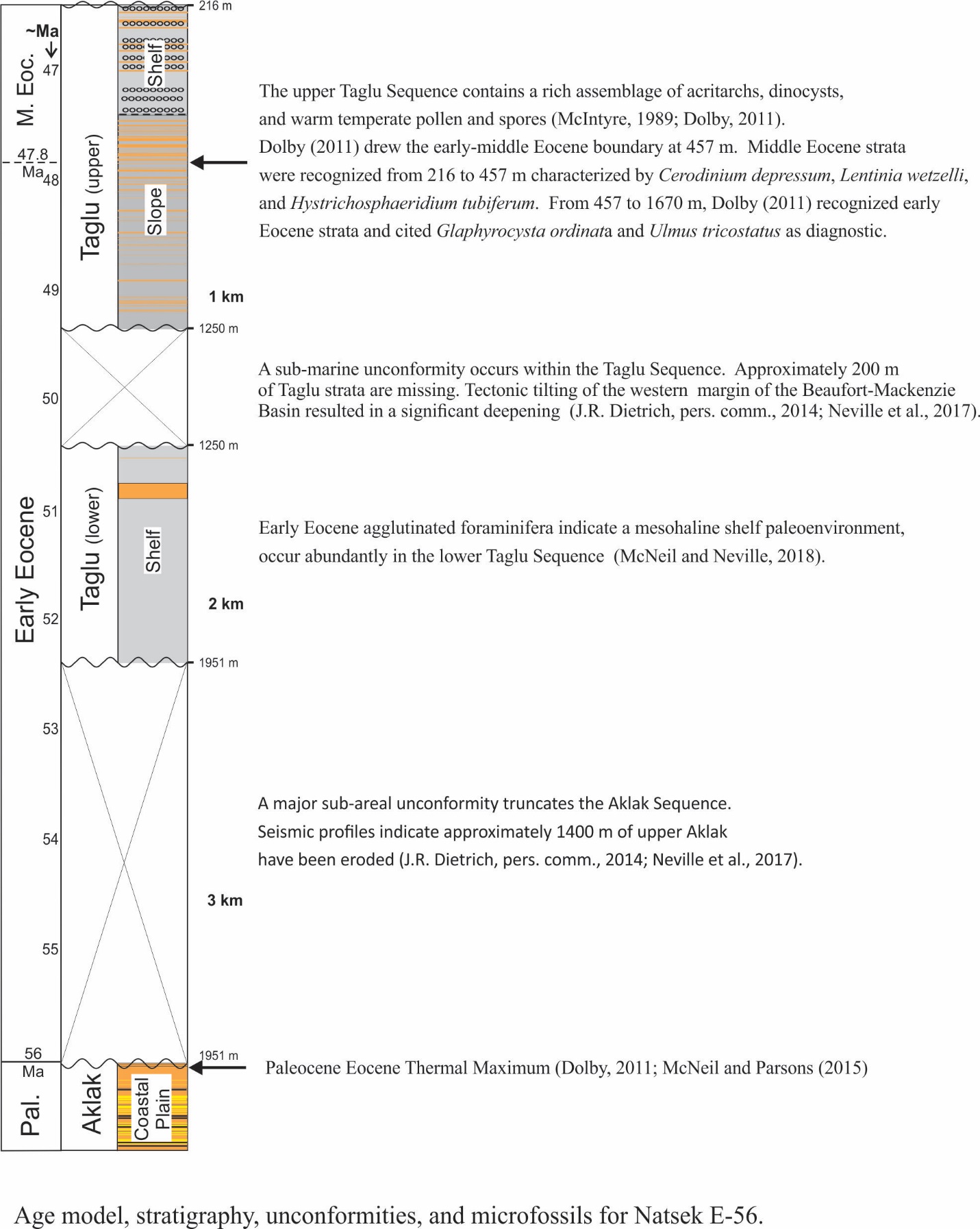


**Fig. S2.** Representative micrographs of the Azolla complex recovered from Natsek E-56. Specimens were immersed in water for photography. The complex consists of three components: megaspores (A), cluster of microsporangia (B), group of microspore massulae? (C).


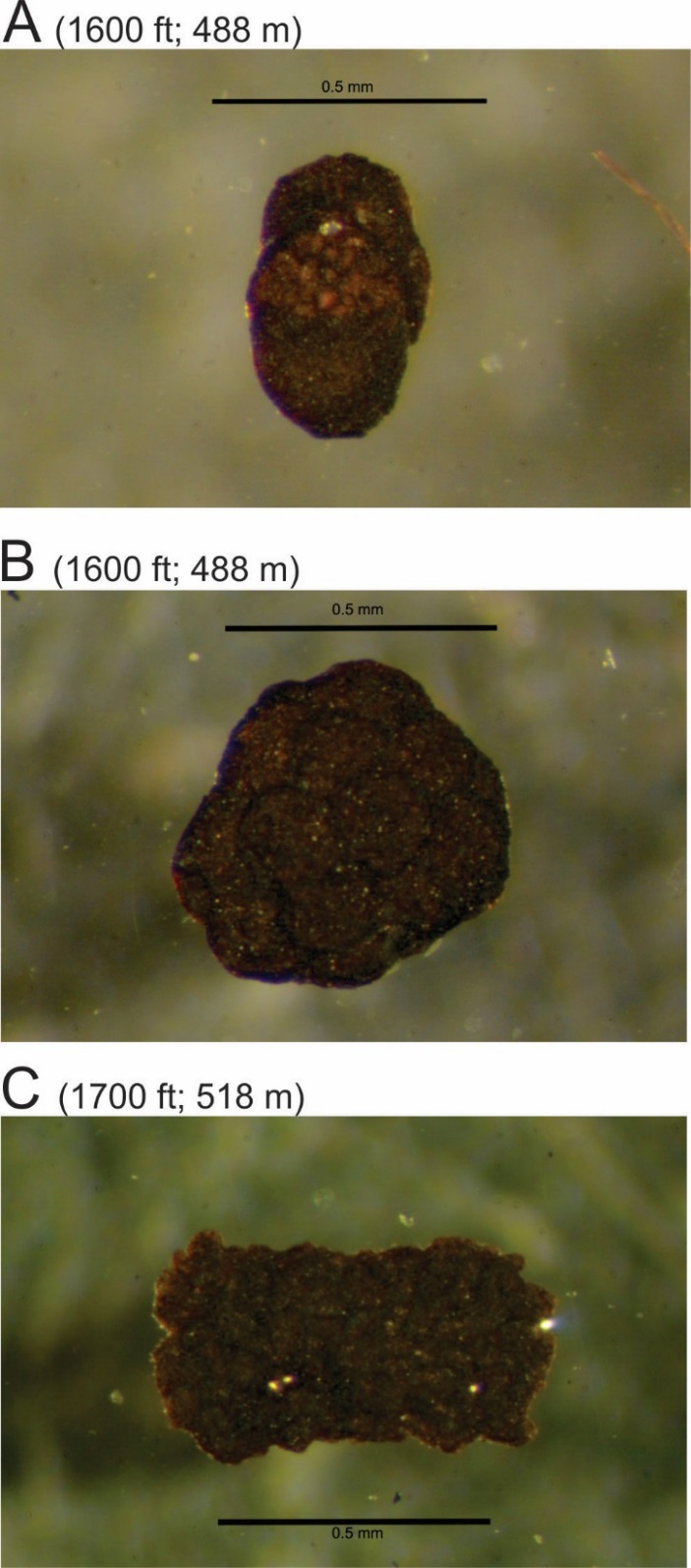


**Fig. S3.** Dinoflagellate counts extrapolated from semi-quantitative data presented by Dolby (2011). Data were originally recorded in five categories: present = 1, rare =2, common = 5, abundant = 15, super abundant = 50. Similarly, *Azolla* miospore counts from Dolby (2011) are shown in the rightmost column.


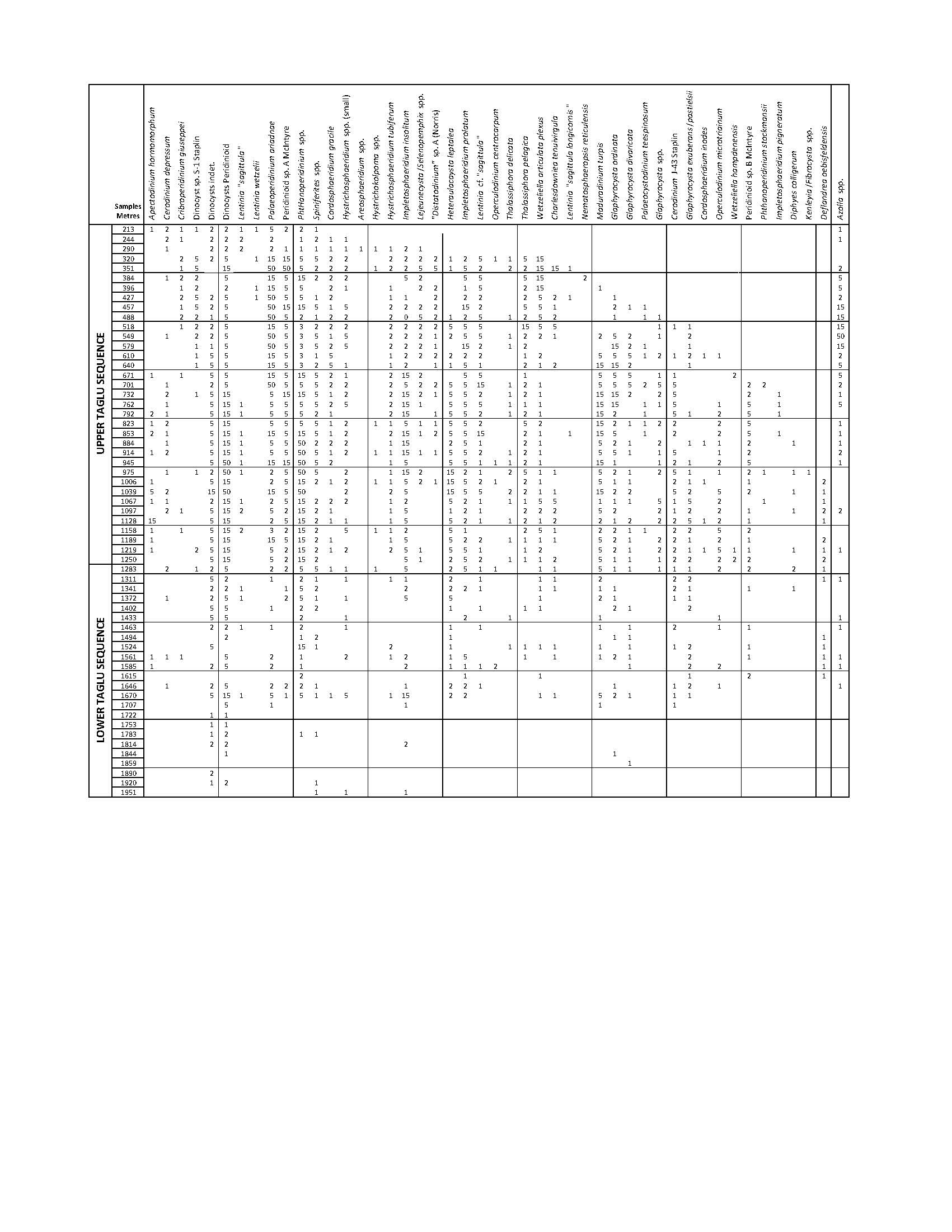


**Fig. S4.** Fungi, pollen, and spores extrapolated from semi-quantitative data presented by Dolby (2011). Data were originally recorded in five categories: present – 1, rare = 2, common = 5, abundant = 15, super abundant = 50.


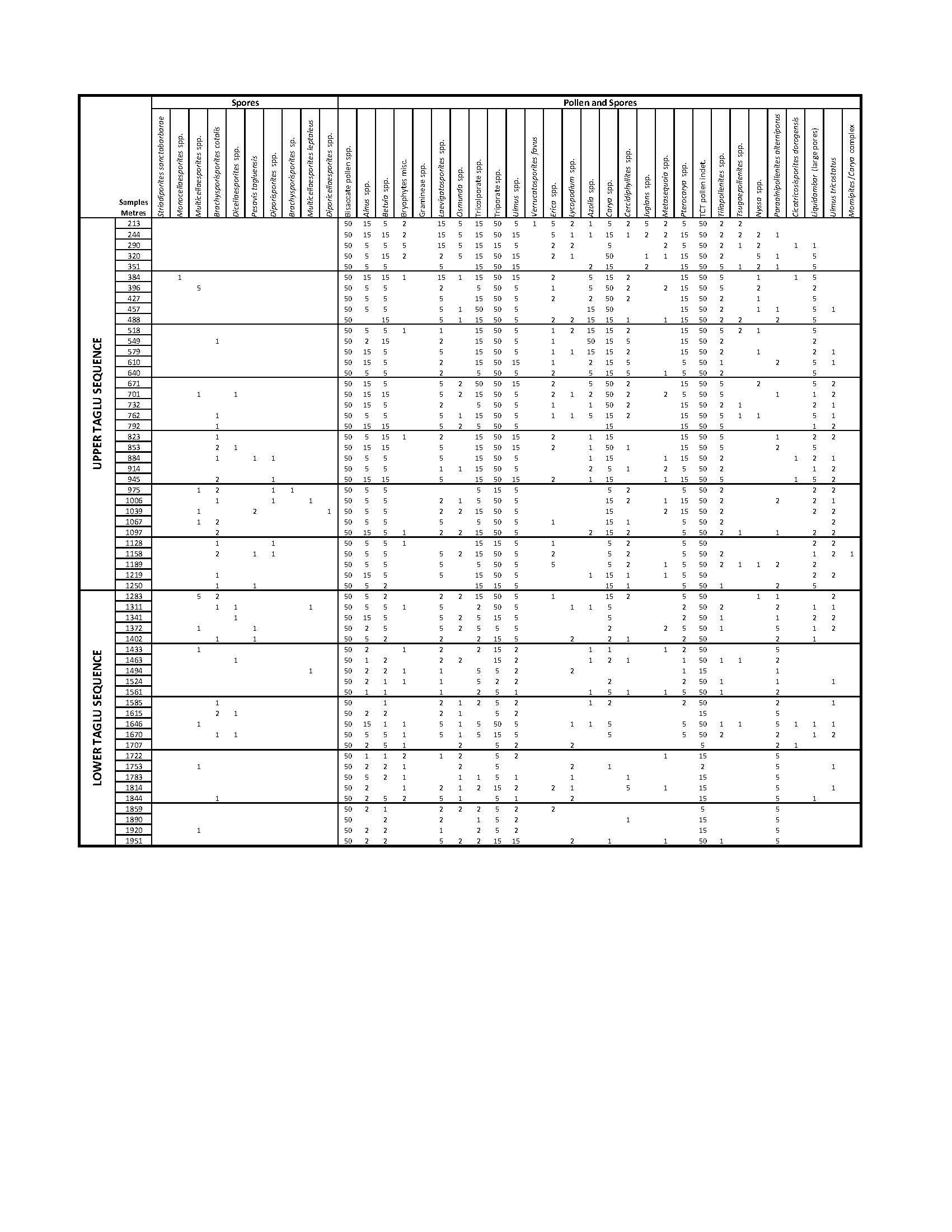


# Table S1

Paleoenvironmental preferences for dinoflagellate cyst genera in the Cretaceous-Cenozoic Labrador-Baffin Seaway (from Nøhr -Hanssen et al., 2016). Genera highlighted in yellow occur also in lower to middle Eocene strata of Natsek E-56. Underlined genera occur in association with *Azolla* in Natsek E-56.

| **Coastal – marginal marine** | **Inner neritic** | **Outer neritic** | **Open Ocean** |
| --- | --- | --- | --- |
| *Eocladopysis*  *Heteraulacacysta*  *Homotryblium*  *Nyktericysta*  *Polysphaeridium*  *Tuberculodinium*  *Vesperopsis* | *Areoligera*  *Cleistosphaeridium*  *Cribroperidinium*  *Deflandrea*  *Dinogymnium*  *Glaphyrocysta*  *Heterosphaeridium*  *Phthanoperidinium*  *Wetzeliella* | *Cerodinium*  *Cleistosphaeridium*  *Cordosphaeridium*  *Hystrichokolpoma*  *Hystrichosphaeridium*  *Operculodinium*  *Phelodinium*  *Spiniferites* | *Cannosphaeropsis*  *Impagidinium*  *Nemostosphaeropsis*  *Pterodinium* |

**Supplementary Information References**

Dolby G. Palynological Analysis of the 152 M – 3520 M TD interval in the Natsek E-56 well, Beaufort Sea. National Energy Board Report; 2011. p. 8.

McIntyre, D.J. *in* Dietrich JR, Dixon, J, McNeil DH, McIntyre DJ, Snowdon LR, Cameron AR. The geology biostratigraphy and organic geochemistry of the Natsek E-56 and Edlok N-56 wells, western Beaufort Sea, Arctic Canada; in Current Research, Part G, Geological Survey of Canada, Paper 89-1G: 133-157, 1989.

McNeil DH, Neville LA, On a grain of sand – a micro-habitat for the opportunistic agglutinated foraminifera Hemisphaerammina apta n.sp, from the early Eocene Arctic Ocean. Journal of Micropalaeontology, 2018, 37: 295-303.

McNeil DH, Parsons MG, The Paleocene-Eocene thermal maximum (PETM) in the Beaufort-Mackenzie Basin – Palynomorphs, carbon isotopes, and benthic foraminiferal turnover. Bulletin of Canadian Petroleum Geology, v. 61, 157-186, 2013.

Neville LA, McNeil DH, Grasby SE, Ardakani OH, Sanei H. Late Paleocene-middle Eocene hydrocarbon source rock potential in the Arctic Beaufort-Mackenzie Basin. *Marine and Petroleum Geology* 2017, **86:** 1082-1091.

Nøhr-Hansen H, Williams GL, Fensome RA. *Biostratigraphic correlation of the western and eastern margins of the Labrador-Baffin Seaway and implications for the regional geology*. Geological Survey of Denmark and Greenland Bulletin 37, 74 p., 2016.
